# Comparison of Two Different Sprint Interval Training Work-to-Rest Ratios on Acute Inflammatory Responses

**DOI:** 10.1186/s40798-016-0044-1

**Published:** 2016-03-01

**Authors:** Christopher R. Harnish, Roy T. Sabo

**Affiliations:** 1 Department of Health and Human Performance, Ferrum College, Ferrum, VA 24088 USA; 2 Department of Biostatistics, Virginia Commonwealth University, Richmond, VA 23298 USA

**Keywords:** SIT, Cytokines, Myokines, Wingate, Tabata

## Abstract

**Background:**

The study aims to compare how work-to-rest ratio (W:R) influences insulin sensitivity (S_i_) and inflammatory responses following one session of sprint interval training (SIT).

**Methods:**

Thirteen men and two women completed a cross-over comparison of two SIT interventions—Tabata (TAB), 10 × 20-s sprints/10-s rest, and Wingate (WIN), 5 × 30-s sprints with 270-s rest. IL-6, IL-10, and TNF-α were assessed at baseline, immediately following, and 1 h after SIT, as well as prior to the 24-h post-exercise oral glucose tolerance tests (OGTTs).

**Results:**

Participants were 23.8 (±3.5) years old and 180.0 (±10.2) cm tall, weighed 78.5 (13.0) kg, and had 16.9 (±6.5) % body fat, with a mean VO_2Peak_ of 42.0 (±7.9) ml kg^−1^ min^−1^. There were no differences in total work (kJ) between TAB (64.7 ± 12.0) and WIN (68.0 ± 15.0). Mean (±95 % CI) S_i_ 24 h changed −2.8 (−5.1, −0.5) from baseline after TAB and −3.9 (−6.9, −0.9) after WIN. Cytokines were measured in pg ml^−1^ and expressed as mean change (±95 % CI). IL-6 increased significantly immediately following SIT for TAB 0.70 (0.23, 1.17), and WIN 1.11 (0.60, 1.62), and remained elevated 1 h post SIT for TAB 1.10 (0.37, 1.83), and WIN 0.95 (0.26, 1.65). IL-10 showed a significant positive change immediately following exercise for TAB 1.53 (0.77, 2.29) and WIN 1.59 (0.58, 2.59). TNF-α also increased immediately both TAB 3.26 (1.57, 4.96) and WIN 3.05 (0.56, 5.54) and was directly proportional to IL-10 (*r* = 0.64, *p* < 0.0001).

**Conclusions:**

W:R did not alter either the inflammatory or metabolic response following SIT nor does SIT improve 24-h S_i_, despite increased levels of IL-10.

## Key Points

- Work-to-rest ratio does not significantly alter the inflammatory or metabolic responses of sprint interval training (SIT).
- An acute bout of SIT does not improve insulin sensitivity in non-obese persons.
- Transient increases in anti-inflammatory cytokines, like IL-6 and IL-10, are associated with insulin sensitivity following SIT.
- There appears to be a strong positive relationship between IL-10 and TNF-α following SIT.

## Background

High-intensity interval training, including sprint interval training (SIT), has been proposed as an effective means for improving both exercise performance and metabolic function [2, 4, 5, 9, 13, 14, 18, 33, 37, 39]. While it has been shown that repeated sessions of SIT using long rest periods (i.e., low work-to-rest ratio) elicit improvements in endurance performance [4, 33] and S_i_ [2, 37, 39], a single bout of SIT appears ineffective for improving S_i_ [3, 30, 38]. In contrast, Whyte et al. [37] showed that a single maximal effort of matched work could improve S_i_, indicating that the mechanisms for metabolic improvements following a single bout of high-intensity exercise require further investigation.

There are many possible mediators of S_i_ and glucose uptake following exercise, including cytokines [10, 11, 26–28, 31]. It is also important to note that while some describe exercise-induced IL-6 release as *anti-inflammatory* [26], others continue to view IL-6 as *pro-inflammatory* [22–24], complicating the interpretation of their impact. For example, endurance exercise has long been viewed as *anti-inflammatory*, resulting in significant increases in circulating cytokines like IL-6 and IL-10, which are believed to improve glucose uptake [21, 26]. In contrast, SIT is viewed as pro-inflammatory with significant increases in IL-6 within 1 h after training [22–24]. More recently, however, Lira et al. [19] have shown that both upper and lower extremity Wingate sprints elicit similar significant increases in IL-10, but not IL-6 immediately following exercise, indicating that high-intensity sprint training may have an anti-inflammatory effect immediately following exercise. Furthermore, unlike endurance exercise, which improves S_i_ after just a single bout, SIT has only been shown to improve S_i_ after two or more weeks of training. Therefore, it is unclear whether inflammatory cytokines are influencing the metabolic changes following a single bout of exercise.

Another important, yet unstudied area, is the effect of work-to-rest ratio (W:R) on the inflammation or S_i_. W:R is an important mediator of metabolic, cardiovascular, and endocrine responses during and after interval and resistance training [8, 15, 16], but data are scarce as to how it influences exercise inflammatory response. Available evidence suggests that a brief bout of maximal sprints with very short rest periods could elicit a significant increase in both IL-6 and IL-10 [11, 25], as well as provide an exercise stimulus more akin to the brief time trial used by Whyte et al. [37]. Thus, we could elucidate whether any single bout of SIT could improve S_i_, and whether inflammatory cytokines are related to such an improvement.

The purpose of this study was to compare the impact of W:R on S_i_ and inflammatory indices. We hypothesized that when matched for total work (kJ), SIT using brief rest periods (W:R = 2:1) would improve S_i_ and associated inflammatory markers more than SIT using long rests (W:R = 1:9).

## Methods

### Participants

A total of 13 men and 2 women were actively recruited for the study. All participants were evaluated for safe exercise participation using an American College of Sports Medicine (ACSM) risk factor assessment and informed of the purposes of the study before signing an informed consent document approved by the Virginia Commonwealth University (VCU) Institutional Review Board. *Inclusion* criteria included men and women between the ages of 18–35 years old who were minimally active—at or below 3 × 30 min of activity/week, and had a body fat ≤25 % for men and 32 % for women. *Exclusion* criteria were any person exceeding the body fat cut off, orthopedic limitations preventing full participation in the study, pre-diabetes or diabetes mellitus, reported hypothyroidism, renal disease, and/or anyone considered *high* risk for exercise participation based on current ACSM clinical guidelines.

### Experimental Protocol

**Fig. 1:**
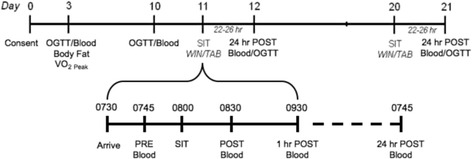
Graphic summary of the experimental design for the study. *OGTT* oral glucose tolerance test, *SIT* sprint interval training, *TAB* Tabata short recovery exercise, *WIN* Wingate long recovery exercise

All experimental procedures were performed in accordance with the ethical standards of the Declaration of Helsinki and approved by the VCU Institutional Review Board. The design (Fig. 1) was similar to previous SIT studies and consisted of a 1-week intra-subject control period. During this period, participants performed baseline (B) and pre-training (PRE) oral glucose tolerance tests (OGTTs). Participants then completed two different acute SIT protocols—*Tabata* and *Wingate*, utilizing a counter balanced cross-over trial design, with each training bout separated by no less than 1 week. Subject 1 was randomly assigned to either Tabata or Wingate first, completing the other session second; each subsequent subject was then assigned to the opposite group first. All exercise took place using a mechanically braked Monark Peak Bike (Monark Exercise AB, Sweden) equipped with an SRM power meter (SRM Service Center, Inc., Colorado Springs, CO). Blood samples (~10 ml) were taken immediately following, and 1 h after each training session, as well as 24 h after exercise; this corresponded to the initial resting sample prior to the post-exercise OGTT. Women participants were tested during the follicular phase of their self-reported menstrual cycle to minimize the impact on cytokine levels.

### Dietary Control

Each subject was asked to complete a 3-day dietary recall form prior to completing any blood analysis. Dietary analysis was performed by a registered dietician for later comparison, and participants were asked to pick 1 day within the recall and repeat those meals the day before each OGTT, recording the meals for those days. Additionally, each participant completed all training sessions after a 12-h fast, including alcohol and caffeine, and they abstained from significant activity 24 h prior to all exercise sessions and OGTTs.

### Preliminary Testing and Evaluation

On the initial assessment day (day 3) and during the OGTT, participants completed body composition analysis using bioelectrical impedance analysis (RJL Quantum IV, RJL Systems, Inc., Clinton Township, MI), where participants lay supine for a period of 20 min to allow body fluids to equilibrate across the body. During this time, small electrodes were placed on the right ankle and wrist. Body composition was then estimated using web-based software (RJL Interactive Online BIA, RJL Systems Inc., Clinton Township, MI). Following the initial OGTT, physiologic testing included bicycle VO_2Peak_ testing. Testing was completed on the SRM equipped Monark bike; power (W) was measured for each stage. VO_2_ and HR were measured continuously using a Parvo OneMax system (Parvo Medics, Salt Lake City, UT) and Polar HR monitor (Polar Electro Inc., New Success, NY), respectively. Participants were instructed to pedal at their preferred cadence throughout testing. The initial workload was set at 1.5 KP with an approximate work rate of 100 W, increasing by 0.5 KP every 2 min until volitional exhaustion was reached, or the subject could not maintain their chosen cadence. Peak power output at VO_2Peak_ was calculated as the highest average 1-min power output achieved during the final stage of testing; this power output was termed *Power at VO*_*2Peak*_*.*

### Exercise Protocols

All SIT sessions began between 0700 and 0900, and each subject’s sessions took place at the same time of the morning. All sprint bouts began with a 10-min unloaded warm-up at ~70 rpm. Participants then pedaled against a resistance equivalent to 7 % (0.07 kg kg^−1^) body mass for Wingate sprints and slightly lower 5 % (0.05 kg kg^−1^) body mass for Tabata. The former resistance has been shown to produce optimal power output and reliable measurement [12], while the latter was shown to be optimal during pilot data work prior to the study. Participants were instructed to pedal as fast as possible for ~2 s before the load is applied and to continue to crank while being provided with vigorous verbal encouragement throughout each sprint. The *Wingate* protocol consisted of a total of five 30-s sprints with approximately 4-min recovery (i.e., very slow unloaded pedaling), while those completing the *Tabata* protocol consisted of ten 20-s sprints with 10-s recovery. The number of intervals performed for each protocol was chosen based on pilot data indicating a close match in total work (kJ).

Peak and mean power (W), as well as total work, were measured and stored using the SRM power meter and downloaded for later analysis using commercially available software (Training Peaks 3.0, Training Peaks, Boulder, CO). Blood lactate samples (5 μl) were measured from the fingertip using a small plastic lancet prior to exercise, immediately following, 1 and 3 min after exercise, and analyzed using a Lactate Scout Analyzer (EKF diagnostic sales GmbH, Barleben/Magdeburg). Each sprint session lasted between 15 and 30 min with warm-up. For descriptive purposes, the relative average power output during each SIT session was expressed a *% Power at VO*_*2Peak*_.

### Blood Analysis

Hemoglobin concentration (g dL^−1^) and hematocrit (%), using the micro-hematocrit method, were measured in duplicate and then used to estimate percentage changes in PV [6]. An indwelling venous catheter was inserted to allow for convenient blood draws. Blood samples (~10 ml) were collected together using gray top sodium fluoride tubes (OGTT) and gold top serum-separator tubes (cytokines) throughout testing and then centrifuged after each session at 4000 rpm for 15 min at 4 °C. Separated plasma was immediately removed and stored in capped 1.5-ml polypropylene tubes frozen at −80 °C until later analysis.

### Oral Glucose Tolerance Tests

OGTTs were completed following insertion of a catheter. Blood (~10 ml) was drawn before, as well as 30, 60, 90, and 120 min after ingestion of a 75 % Glucola drink (Fisher Science Inc., Philadelphia, PA). Plasma glucose concentrations (mg dl^−1^) were measured using the auto-analyzer glucose oxidase method, while plasma insulin concentrations (mU l^−1^) were determined by ELISA (R&D Systems, Inc, Minneapolis, MN). The coefficient of variation (CV) for baseline *Cederholm* S_i_ was 4.8 %.

### Inflammatory Markers

Inflammatory markers of interest included IL-6 (IL-6 B), IL-10 (IL-10 B), and TNF-α (TNF-α B) measured during baseline testing periods, as well as following each bout of SIT based on the time periods reported in prior research [22–24]. At baseline, samples were analyzed from the blood taken at minute 0. On SIT days, 10 ml of blood was taken prior to, immediately following exercise (P), and 1 h later (P 1). A final cytokine measurement was taken prior to the OGTT ~24 h after the SIT bout (P 24). Plasma concentrations of IL- 6, IL-10, and TNF-α were determined using interleukin-specific Humakine ELISA kits (R&D Systems, Minneapolis, MN), each completed according to manufacturer’s instructions. Coefficients of variation (CV) for IL-6, IL-10, and TNF-α were 9.9, 6.1, and 6.6 %, respectively.

### Statistical Analysis

Data analysis was performed using commercially available software (Jump 13.0, SAS Institute Inc, Cary, NC). During the design process, power analyses run for cytokines and S_i_ estimated that an *N* of 15 provides a power of 0.85. All data are presented as means ± SD. Area under the curve (AUC) was calculated using the trapezoidal rule, while the Cederholm index, which represents peripheral S_i_, was calculated using the formula:

$$ \frac{\mathrm{Cederholm}\ {\mathrm{S}}_{\mathrm{i}} = 75,000 + \left({G}_0-{G}_{120}\right) \times 1.15 \times 180 \times 0.19 \times \mathrm{B}\mathrm{W}/120 \times {G}_{\mathrm{mean}} \times \log\ \left({I}_{\mathrm{mean}}\right)}{1000} $$

BW is the body weight, *G*_0_ and *G*_120_ are plasma glucose concentration at 0 and 120 min (mmol l^−1^), and *I*_mean_ and *G*_mean_ are the mean insulin (mU l^−1^) and glucose (mmol l^−1^) concentrations during the OGTT.

All exercise responses (S_i_, glucose AUC, insulin AUC, and cytokines) are reported as absolute values and changes from baseline. Data were analyzed using absolute change responses from baseline ± 95 % confidence intervals; 95 % CI changes that failed to cross 0 (i.e., 0 change) were considered significant. Dependent *t* tests were run to compare change in S_i_ following *Tabata* and *Wingate* SIT from baseline and at 24 h. IL-6, IL-10, and TNF-α were compared between SIT groups using a similar repeated measures ANOVA model, though with a four-level time indicator (baseline, post, 1 h, 24 h). Finally, Pearson’s correlation coefficients were calculated to examine the relationships between S_i_ and changes in cytokine response.

## Results

**Fig. 2:**
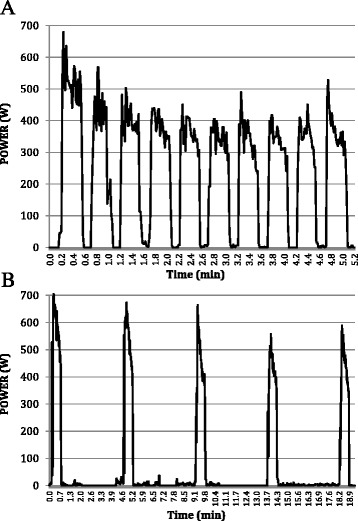
Sample sprint interval training session of one participant for Tabata (**a**) and Wingate (**b**)

Fifteen participants completed both sprint sessions, with one subject unable to complete a 24-h follow-up after the Wingate session due to inclement weather. Participants were 23.8 (±3.5) years old and 180.0 (±10.2) cm tall, weighed 78.5 (13.0) kg, and had 16.9 (±6.5) % body fat, with a mean VO_2Peak_ of 42.0 (±7.9) ml kg^−1^ min^−1^ at 237.0 (±56.6) W. Dietary analysis indicated our participants consumed a diet consisting of 2077.5 ± 132.3 kcal from 81.9 ± 8.0 g of fat, 243.2 ± 14.4 g of carbohydrate, and 93.2 ± 6.8 g of protein, and were without any remarkable findings. Table 1 provides a summary comparison of SIT session variables. The % Power at VO_2Peak_, was significantly higher (*p* < 0.0001) during WIN (196.8 ± 24.4 %) compared to TAB (95.6 ± 8.9 %). While there were no differences in total kJ (*p* = 0.5152) or blood lactate (*p* = 0.8307) between SIT sessions, HR was significantly lower (*p* = 0.0272) during Wingate sessions. Figure 2 depicts sample SIT sessions for one subject. Overall responses for men and women overlapped and trended similarly and therefore were analyzed together.

**Table 1:** Comparison of *Tabata* and *Wingate* protocols and exercise session data

	Tabata	Wingate	*p* value
Mean power (W)	223.2 (40.9)^a^	457.8 (84.1)^a^	<0.0001
% Power at VO_2Peak_	95.6 (8.9)^a^	196.8 (24.4)^a^	<0.0001
Work (kJ)	64.7 (12.0)	68.0 (15.0)	0.515
BLC (mM)	12.8 (2.6)	12.6 (2.6)	0.831
HR (bpm)	180.7 (9.5)	173.9 (9.0)^a^	0.0272

Data are presented as means (±SD)

^a^Significant (*p* < 0.05) difference between interventions

### Insulin Sensitivity

Baseline S_i_ for all participants was 75.9 (±1.0) mg *I*^2^ mM^−1^ mU^−1^ min^−1^. Table 2 summarizes the S_i_, glucose AUC, and insulin AUC data for both SIT sessions using an intent-to-treat analysis. As noted above, one subject was unable to complete a 24-h post testing, including OGTT, after WIN training. The data indicate that S_i_ was significantly depressed after both TAB, −2.8 (−5.1, −0.5), and WIN, −3.9 (−6.9, −0.9), but no differences between sprint interventions (*p* = 0.6723), and AUC for neither glucose nor insulin were significantly impacted (see Table 2).

**Table 2:** Comparison of baseline (mean ± SD) and mean (±95 % CI) changes for glucose, insulin, and cytokine measures

Measure		Change	Change	Group comparison
	Baseline	Tabata	Wingate	
Insulin sensitivity	75.9 (3.2)	−2.8 (−5.1, −0.5)^a^	−3.9 (−6.9, −0.9)^a^	0.5115
Glucose AUC	444.1 (78.9)	−17.8 (−12.90, 48.4)	−14.8 (−35.6, 27.8)	0.3228
Insulin AUC	91.9 (55.2)	−12.4 (−6.5, 31.2)	−4.7 (−17.45, 19.0)	0.3494
Cytokine response				
IL-6 (pg ml^−1^)	0.85 (0.48)			
Post 0		0.70 (0.23, 1.17)^b^	1.11 (0.60, 1.62)^b^	0.9091
Post 1 h		1.10 (0.37,1.83)^b^	0.95 (0.26, 1.65)^b^	0.9991
Post 24 h		−0.13 (−0.38, 0.13)	0.47 (−0.34, 1.28)	0.6706
IL-10 (pg ml^−1^)	3.69 (1.46)			
Post 0		1.53 (0.77, 2.29)^b^	1.59 (0.58, 2.59)^b^	1.0000
Post 1 h		−0.4091(−2.050, 1.23)	0.31 (−0.56, 1.18)	0.8771
Post 24 h		−0.56 (−1.37, 0.23)	−0.03 (−0.79, 0.73)	0.9538
TNF-α (pg ml^−1^)	3.96 (3.45)			
Post 0		3.26 (1.57, 4.96)	3.05 (0.56, 5.54)	1.0000
Post 1 h		−1.41 (−3.93, 1.11)	−0.94 (−3.22, 1.35)	0.9993
Post 24 h		−0.59 (−2.72, 1.53)	0.08 (−2.03, 2.20)	0.9973

Group comparison *p* values indicate there were no differences between sprint groups

^a^95 % CI changes that failed to cross 0 (i.e., 0 change) were considered significant with a significant decrease above baseline

^b^95 % CI changes that failed to cross 0 (i.e., 0 change) were considered significant with a significant increase above baseline

### Inflammatory Cytokine Response

Changes in glucose, insulin, and cytokines are summarized in Table 2. Plasma cytokine levels for IL-6, IL-10, and TNF-α were measured in all 15 participants during pre-OGTT (baseline) and TAB test sessions, where 14 participants of 15 participants were measured 24 h after WIN. IL-6 increased significantly immediately following SIT for TAB 0.70 (0.23, 1.17), and WIN 1.11 (0.60, 1.62), and remained elevated 1-h post SIT for TAB 1.10 (0.37, 1.83), and WIN 0.95 (0.26, 1.65). IL-10 showed a significant positive change immediately following exercise for TAB 1.53 (0.77, 2.29) and WIN 1.59 (0.58, 2.59). TNF-α also increased immediately both TAB 3.26 (1.57, 4.96) and WIN 3.05 (0.56, 5.54). All cytokines returned to baseline levels 24 h after exercise. The relative anti-inflammatory response, expressed as a ratio between IL-10 and TNF-α did not change significantly for any time period (*p* = 1.0) and ranged from 0.62 to a peak of 0.88 and 0.79 immediately following TAB and WIN, respectively. Results of all significant Pearson correlations are summarized in Table 3. IL-6 was not related to either IL-10 or TNF- α but showed a strong inverse relationship at baseline with S_i_. IL-10 and TNF-α were positively related (*r* = 0.64, *p* < 0.001) overall, as well as immediately following SIT.

**Table 3:** Summary of significant Pearson correlation coefficients

Independent variable	Dependent variable	*r*	*p* value
Baseline IL-6	Baseline S_i_	*r* = −0.65	
TNF-α	IL-10	*r* = 0.64	<0.0001
Tabata			
TNF-α P	IL-10	*r* = 0.83	0.0027
TNF-α P 24	IL-10	*r* = 0.76	0.0472
Wingate			
TNF-α P	IL-10	*r* = 0.93	0.0001

No other significant relationships were observed

*B* baseline, *P* immediately post exercise, *P 24* 24 h post exercise

## Discussion

The purpose of this study was to compare how W:R influences inflammatory and metabolic responses following a single bout of SIT. It was believed that when matched for total work (kJ), SIT using brief rest periods (W:R = 2:1) would elicit a greater improvement in S_i_ proportional to a higher cytokine response than SIT using long rests (W:R = 1:9). However, our data indicate that both SIT sessions depressed S_i_ 24 h after exercise. In contrast, both sessions increased IL-6, IL-10, and TNF-α for up to 1 h after exercise. While IL-10 and TNF-α release appear to be directly proportional to each other following SIT, their impact on S_i_ is unclear.

### SIT and S_i_

A major finding of this research was that neither TAB nor WIN SIT improved S_i_ P 24, indicating that W:R does not influence S_i_ in healthy young adults. In the present study, S_i_ was actually significantly decreased by 5 % following WIN, while 5 min of TAB decreased S_i_, by 3.6 % 24 h after exercise. This finding was consistent among participants in both SIT trials, with only 3 of 15 participants actually improving S_i_ following TAB. Close examination of these data show that 2 of 15 participants had 15 % or greater decrease in S_i_ following WIN. Interestingly, the (male) subject with the largest decrease in S_i_, ~15 % following TAB and 20 % following WIN, also had the highest body fat at 24.8 %. However, no other differences, including diet, were noted between participants, and removal of these outliers did not reverse the trend toward reduced S_i_.

The lack of improvement following TAB was unexpected because total work was similar to that of the extended sprint reported by Whyte et al. [38]. In that study, subjects performed two interventions, four WIN intervals, and a maximal ~200-s extended sprint (ES), both resulting in ~62 kJ. S_i_ improved significantly 24 h after the ES, but not WIN. In fact, a single bout of WIN has failed to improve S_i_ in other studies as well [3, 30]. These researchers suggested that the key factor for acute improvements in S_i_ may be ATP turnover [36], which would be maximal during the ~200-s continuous time trial used in their study. It was this premise that influenced our choice of the *Tabata* SIT intervention.

As reported by Tabata et al. [34, 35], 4 min of the 20-s work to 10-s rest ratio maximally stimulated oxygen consumption and anaerobic capacity; peak VO_2_ in the final 10 s of the 4-min Tabata was similar to their participants’ VO_2max_, as was the accumulated O_2_ deficit. In order to match work in our study, our TAB SIT session lasted an additional 50 s (i.e., two 20-s sprints), with a total duration exceeding Whyte’s ES but likely maximally stimulating mitochondrial ATP production. Therefore, work and ATP turnover may not be critical factors at play. Of particular note, however, Whyte et al. [38] enrolled overweight and obese male participants, while we studied healthy young adults, who were relatively lean (body fat % = 16.9). Not surprisingly, the outlier showing the greatest drop in S_i_ following TAB also had the highest body fat at nearly 25 %. Therefore, overweight and obese individuals may be more responsive to single bouts of SIT than lean individuals. Changes in S_i_ following a single session of SIT are more complex and warrant further investigation.

### SIT Impact on the Inflammatory Response

Another major finding of this study was that W:R does not alter the effect on inflammatory cytokine release following SIT, as there were no differences between SIT groups for any cytokine measured. In addition, we noted that IL-10 release was not dependent on IL-6 but that increases in IL-10 immediately following SIT were directly proportional to TNF-α (*r* = 0.64, *p* < 0.001), supporting, in part, the findings of Lira et al. [19], but not other SIT research. For example, prior research by Meckel et al. [22, 23] and Nemet et al. [24] reported that running sprint exercise significantly increased IL-6 1 h after exercise but did not influence IL-10 [24] in trained men and women. In contrast, Brestoff et al. [3] showed that a session of five Wingate sprints (1:9 W:R) did not alter IL-6 or TNF-α release after exercise in recreationally active men and women. Most recently, however, Lira et al. [19] reported a similar significant increase in IL-10 immediately following lower extremity Wingate sprints, but not upper extremity sprints; they also failed to show an increase in TNF-α.

A major premise of cytokine release following endurance exercise is that IL-6 is released directly from the muscle, making it a myokine [26], and that this release from the muscle influences its anti-inflammatory role [27, 28]. Further, IL-6 is believed to be a stimulant to IL-10 release, which is a known inhibitor of TNF-α [1, 7, 32]. These data suggest that SIT, regardless of W:R, results in a nearly 60 % increase in TNF-α immediately following exercise, as well as a similar increase in IL-10, which leads to a consistent yet insignificant increase in the IL-10-to-TNF-α ratio. This finding is important for two reasons. First, it supports recently published data indicating that IL-10 increases following intense exercise independent of IL-6. Second, it suggests that IL-10 may increase in response to TNF-α. The latter is supported by our data showing that both IL-10 and TNF-α increased significantly immediately after both TAB and WIN, which is then followed by a significant decrease in TNF-α 1 h after exercise. Furthermore, IL-10 levels were strongly related (*r* = 0.87, *p* < 0.005) to TNF-α immediately following both SIT sessions, which is not surprising, as IL-10 has been shown to be a potent mediator of TNF-α [1, 7]. A pro-inflammatory response, though transient, may be an important stimulus for long-term adaptation [5], similar to the role reactive oxygen species, like H_2_O_2_, and lactate accumulation, play in mitochondrial biogenesis [17, 20]. It is also possible it simply reflects increased glycogenolysis and lipolysis [21].

### SIT: Inflammation and Glucose Regulation

Presently, our understanding of cytokine release following SIT is limited, and any relationship to acute improvement in S_i_ is largely based on endurance exercise studies [10, 26, 28, 32]. Our data indicate that SIT does not have an acute impact on IL-6 release, nor does acute SIT influence on S_i_, regardless of W:R. Additionally, there was no relationship between cytokine release and S_i_, suggesting that inflammatory cytokines do not play a significant role in the improved S_i_ seen in SIT studies of at least 2 weeks [2, 30, 37, 39]. Newly published data indicate a disruption in the sarcoplasmic reticulum (SR) following a single bout of SIT [29]. The disruption in the SR has been postulated as the trigger for reported improvements in the chronic endurance performance and metabolic function following two or more weeks of SIT [4, 29]. For example, Coffey and Hawley [5] outlined four distinct signals for mitochondrial biogenesis and improved glucose regulation, including mechanical stretch, increased intramuscular calcium concentration, reduced muscle ATP concentrations, and an increase in ROS, or other disruptions to muscle homeostasis. While Place et al. [29] reported the latter two signals in their recent work, only the last would support the role of inflammatory cytokines in this process. Nonetheless, far more work is needed before direct conclusions can be drawn.

These results add to a growing body of literature elucidating the inflammatory response following SIT and its possible role in the chronic training improvements reported from a variety of sprint protocols. While intriguing, our results represent a relatively healthy population of college-age adults. The fact that these data seem to contrast those of Meckel et al. [22, 23], who studied sprint training in elite athletes during run SIT, indicates that population characteristics, fitness level, and mode likely influence inflammation in SIT. The results in the present investigation demonstrate the need for examining a number of mechanisms in various populations to better understand the possible health benefits of SIT.

## Conclusions

In conclusion, our data indicate that W:R does not significantly alter the metabolic and inflammatory responses following SIT of similar work. Moreover, it appears that despite rapid increases in IL-6 and IL-10, SIT actually suppressed S_i_ 24 h after exercise. The rapid peaks in TNF-α and IL-10 are supported by data published for Wingate SIT [19] and are more akin to the release pattern seen during sepsis [1, 7, 11]. It is unclear, however, whether the cytokine release pattern represents a pro-inflammatory environment or merely relates to increased substrate mobilization. Nonetheless, the post-exercise environment may be an important stimulus for long-term positive adaption to high-intensity sprint and interval training regimens.
